# Quality of life reported by patients with ecchymosis following total knee arthroplasty

**DOI:** 10.3389/fsurg.2025.1515378

**Published:** 2025-03-13

**Authors:** Zhibing Gong, Hanglin Qiu, Huantang Zhang, Yanyan Xu, Rongkai Wu, Qianjin Zhang, Hanghui Lin, Zhaoke Wu, Fudong Xu, Zhikun Zhuang, Changyu Huang

**Affiliations:** Department of Orthopaedic Surgery, Quanzhou Orthopedic-Traumatological Hospital, Quanzhou, China

**Keywords:** total knee arthroplasty, quality of life, ecchymosis, patient-reported outcome measures, risk factors

## Abstract

**Objectives:**

To explore the early quality of life (QOL), function, and pain of patients with ecchymosis after total knee arthroplasty (TKA) using Patient-Reported Outcome Measures (PROMs), and to investigate the incidence of post-TKA ecchymosis and its potential risk factors under anticoagulant therapy.

**Methods:**

This single-center observational study included patients who underwent TKA at our center from June 2022 to June 2023. Data on demographic information, surgical details, pre-operative and post-operative laboratory results, imaging data, etc., were collected. Patients were divided into two groups based on the presence or absence of ecchymosis after TKA: the ecchymosis group and the non-ecchymosis group. Patients' QOL postoperatively was assessed using Visual Analog Scale (VAS), Hospital for Special Surgery (HSS) score, and Rand 36-Item Short Form Health Survey (SF-36). A binary logistic regression model was employed to analyze the risk factors for post-TKA ecchymosis.

**Results:**

A total of 138 participants were included, 15 males and 123 females, with a mean age of 67.91 ± 7.24 years and BMI of 25.57 ± 3.85 kg/m^2^. There were 60 cases in the ecchymosis group and 78 cases in the non-ecchymosis group, resulting in an incidence of post-TKA ecchymosis of 43.48%. The ecchymosis group demonstrated more significant pain and poorer joint function postoperatively, with significantly lower scores in the SF-36 dimensions and 7 subdomains compared to the non-ecchymosis group. Regression analysis suggested that age may be a risk factor for post-TKA ecchymosis.

**Conclusions:**

The incidence of ecchymosis after TKA is high under anticoagulant therapy, leading to increased pain sensitivity, decreased function, and quality of life in patients. Age may be a risk factor for early post-TKA ecchymosis. Incorporating information on ecchymosis into preoperative education and providing appropriate psychological interventions for patients experiencing ecchymosis may be necessary.

## Introduction

Total knee arthroplasty (TKA) has become the most common and effective treatment for end-stage knee osteoarthritis (KOA) due to its ability to relieve pain and restore function (1). In the United States, based on data from 2019, it is projected that the number of TKAs will increase by 139% by 2040 and by 469% by 2060 (2). Although TKA patients generally have better mid-to-long-term quality of life scores post-surgery, the 5-year satisfaction rate is only 75%, with post-operative complications such as infection, thromboembolism, pain, and psychological or emotional factors considered as important factors influencing satisfaction (3).

Venous thromboembolism (VTE) and pulmonary embolism (PE) are rare but life-threatening complications following TKA. With the routine use of anticoagulant therapy by clinicians, the incidence of postoperative PE after TKA has been controlled within the range of 0.5% to 1% (4). However, standard anticoagulation therapy carries an increased risk of bleeding (5). Ecchymosis, a manifestation of bleeding, is a common symptom post-TKA, with an incidence rate of up to 33% (6). Ecchymosis near the incision site may lead to local infection and increased psychological stress, which can also impact post-TKA functional recovery and quality of life (7, 8).

Despite the high incidence rate, clinicians seem to still underestimate the importance of ecchymosis, as generally, ecchymosis at the TKA incision site can self-resolve without the need for specific interventions, even without discontinuing anticoagulants unless there is a clear risk of major bleeding. However, this visible ecchymosis can be distressing for patients, causing significant anxiety that may affect their quality of life (QOL), and even raise doubts about the success of the surgery in the early post-TKA period (9). Unfortunately, there is limited reporting on the quality of life, pain levels, joint function, and other aspects in patients who develop ecchymosis following TKA. Patient-reported outcome measures (PROMs) provide a standardized evaluation of patients' health status and healthcare experiences directly from patients, offering valuable insights for clinicians to understand the impact of treatment on patients' QOL and potentially influencing treatment decisions (10).

Therefore, this study was conducted with the aim to: (1) investigate the early impact of post-TKA ecchymosis on patients’ QOL, function, pain, etc., using PROMs; and (2) examine the incidence rate of post-TKA ecchymosis and potential risk factors under routine anticoagulant therapy.

## Methods

### Participants

This single-center observational study has been approved by our institutional review board (Approval Number: IRB of QZOTH-2020-002-32) and obtained informed consent from all participants. All participants were recruited from our institution, a tertiary-care orthopedic center. From June 2022 to June 2023, patients undergoing TKA at our center were consecutively enrolled in the study. Inclusion criteria were patients aged 18 years and above requiring primary TKA. Exclusion criteria included: (1) patients with a history of intra-articular or periarticular skin infection, tumors, or joint tuberculosis; (2) patients requiring long-term anticoagulant therapy due to reasons such as prior vascular stent implantation.

### Operation and treatment

All participants underwent surgery under general anesthesia, with the initial TKA performed using the medial parapatellar approach by the same surgical team. Intraoperatively, a tourniquet was routinely applied, and a regional injection was administered around the joint with 60 ml of normal saline containing betamethasone (10 mg) and ropivacaine (100 mg). After closure of the joint capsule, tranexamic acid (2 g tranexamic acid + 20 ml normal saline) was injected for hemostasis, without the use of a drainage tube. Postoperatively, all patients received parecoxib for pain management (40 mg, q12 h, intravenously) and continued oral sustained-release diclofenac sodium tablets (75 mg) for 2 weeks post-discharge. Rivaroxaban anticoagulation therapy was initiated on postoperative day 1 (10 mg, once daily, orally) and continued for 14 days postoperatively. The decision to adjust the anticoagulant regimen for patients with ecchymosis was made by the surgical team, and the researchers did not intervene in the clinical treatment plan.

### Data collection

Demographic information, surgical details, preoperative and postoperative laboratory data, and imaging data were collected, including patient age, gender, body mass index (BMI), surgical side, smoking and drinking history, hypertension, diabetes, osteoporosis, operation time, intraoperative bleeding, tourniquet time, coagulation time, fibrinogen levels, postoperative hemoglobin (HGB) decrease on the first and third days, international normalized ratio (INR), and imaging data. Patients were categorized into two groups based on the presence or absence of ecchymosis post-TKA. The area of ecchymosis was measured using a transparent soft card with a scale, a pragmatic and clinically feasible method. Parameters such as the onset time, location, and resolution time of the ecchymosis were also recorded. While this method is practical for routine clinical use, future studies could explore more precise techniques, such as digital imaging or photogrammetry, to enhance measurement accuracy.

A trained investigator conducted weekly telephone questionnaire surveys from postoperative week 1 to week 4, unaware of whether patients had ecchymosis. Patients reported visual analogue scale (VAS) scores and Hospital for Special Surgery (HSS) scores each week, with a QOL score collected at week 4. The Rand 36-Item Short Form Health Survey (SF-36) was used to assess postoperative QOL in patients, as this standardized tool has been shown to be effective in assessing patients undergoing total joint arthroplasty (11, 12). Scores were calculated for 8 independent scales across 2 dimensions (physical and mental health). SF-36 scores were transformed to a 0–100 scale (0 = worst, 100 = best), a conversion method previously applied in TKA studies (13). Based on previous research, the Minimum Clinically Important Differences (MCID) are defined as a 5-point change (14).

### Statistics

Descriptive statistics were used to present parameters with means ± standard deviations (*X* ± *S*) or medians with interquartile ranges (median, IQR). Statistical significance of various parameters was analyzed using *t*-tests, rank-sum tests, or chi-square tests. Changes in HGB, VAS, and HSS scores were plotted using GraphPad Prism 9.0.0 (GraphPad Software, USA). A binary logistic regression model was used to analyze potential risk factors for ecchymosis. The “B” coefficient represents the relationship between each predictor and the log odds of developing ecchymosis. Specifically, a positive coefficient implies that as the independent variable increases, the likelihood of developing ecchymosis also increases; whereas a negative coefficient suggests a decrease in likelihood. The binary logistic regression analysis allows for a direct assessment of risk factors for event occurrence and is widely recognized. A two-tailed *P*-value less than 0.05 was considered statistically significant. Statistical analysis was performed using SPSS 26.0 (IBM, Armonk, New York, USA).

## Results

**Figure 1:**
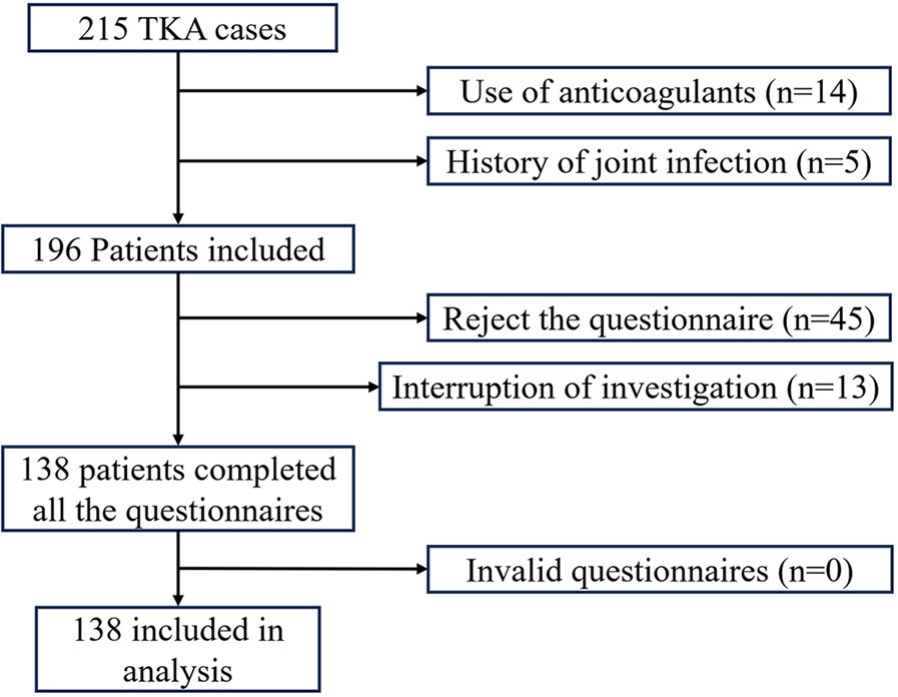
Flow diagram of the current study.

A total of 215 patients underwent TKA, with exclusion of patients on long-term anticoagulant therapy (*n* = 14), a history of joint infection (*n* = 5), those who refused telephone follow-up (*n* = 45), and individuals who did not complete all telephone visits (*n* = 13). Finally, 138 participants were included in the study, as illustrated in the research flow diagram in Figure 1. Of these participants, there were 8 cases of traumatic arthritis, 8 cases of rheumatoid arthritis, and 122 cases of osteoarthritis. There were 15 male and 123 female patients, with a mean age of 67.91 ± 7.24 years (range: 51–84), 63 cases involving the left knee and 75 cases involving the right knee, and a mean BMI of 25.57 ± 3.85 kg/m^2^. The study included 60 patients with ecchymosis and 78 patients without ecchymosis, with no significant differences observed between the two groups in demographic, surgical, preoperative laboratory, and imaging data (Table 1).

**Table 1:** Demographics of participants.

Variable	Total (*n* = 138)	Ecchymosis (*n* = 60)	Non-ecchymosis (*n* = 78)	*p*-value
Age (years, $\bar{X}\pm S$)	67.91 ± 7.24	69.12 ± 6.40	66.97 ± 7.73	0.09^a^
Gender (*n*)				0.42^c^
Male	15	8	7	
Female	123	52	71	
BMI (kg/m^2^, $\bar{X}\pm S$)	25.57 ± 3.85	26.04 ± 3.65	25.20 ± 3.98	0.20^a^
Surgical side (left/right)	63/75	23/37	40/38	0.13^c^
Ever-smoker (*n*)	20	9	11	0.89^c^
Alcohol consumption (*n*)	36	16	20	0.80^c^
Diabetes (*n*)	37	15	22	0.67^c^
Hypertension (*n*)	39	22	18	0.08^c^
K-L score (median, IQR)	4 (4, 4)	4 (4, 4)	4 (4, 4)	0.12^b^
Osteoporosis (n)	57	19	38	0.04^c^
Operative time (min, $\bar{X}\pm S$)	74.29 ± 15.99	76.07 ± 15.58	72.92 ± 16.26	0.25^a^
Tourniquet time (min, $\bar{X}\pm S$)	64.96 ± 11.99	66.67 ± 12.08	63.65 ± 11.82	0.14^a^
Intraoperative blood loss (ml, $\bar{X}\pm S$)	86.67 ± 38.40	85 ± 41.48	87.95 ± 36.09	0.66^a^
Preoperative HGB (g/L, $\bar{X}\pm S$)	131.19 ± 12.38	131.03 ± 11.99	131.31 ± 12.75	0.90^a^
Preoperative INR ($\bar{X}\pm S$)	0.91 ± 0.06	0.91 ± 0.05	0.92 ± 0.06	0.38^a^
Preoperative PT (S, $\bar{X}\pm S$)	10.78 ± 1.15	10.59 ± 1.46	10.92 ± 0.82	0.09^a^
Preoperative FIB (median, IQR)	3.81 ± 2.77	3.87 ± 3.91	3.77 ± 1.40	0.84^a^

BMI, body mass index; K–L, Kellgren–Lawrence; HGB, hemoglobin; INR, international normalized ratio; PT, prothrombin time; FIB, fibrinogen.

a Independent-samples *t*-test.

b Mann–Whitney *U* test.

c Chi-squared.

**Figure 2:**
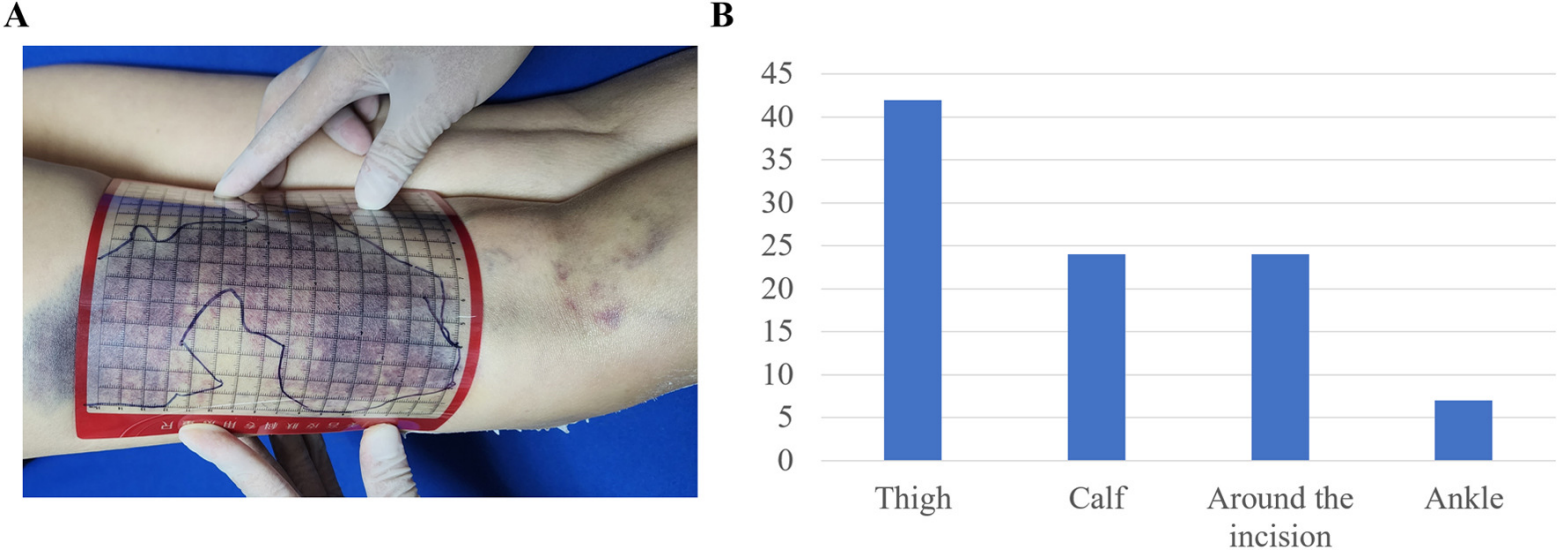
Measurement method and distribution of ecchymosis. **(A)** Measurement method of ecchymosis; **(B)** distribution of ecchymosis locations.

The incidence rate of post-TKA ecchymosis was 43.48% (60/138), with a median time of ecchymosis appearance at 3 days postoperatively (IQR: 3, 4). The average area of ecchymosis (Figure 2A) was 190.87 ± 25.75 cm^2^. Locations of ecchymosis included the thigh (*n* = 42), calf (*n* = 24), around the incision site (*n* = 24), and ankle (*n* = 7) (Figure 2B). After 21 days postoperatively, 73.33% of patients showed disappearance of ecchymosis, and by day 28 postoperatively, 93.33% of patients exhibited resolution of ecchymosis.

**Figure 3:**
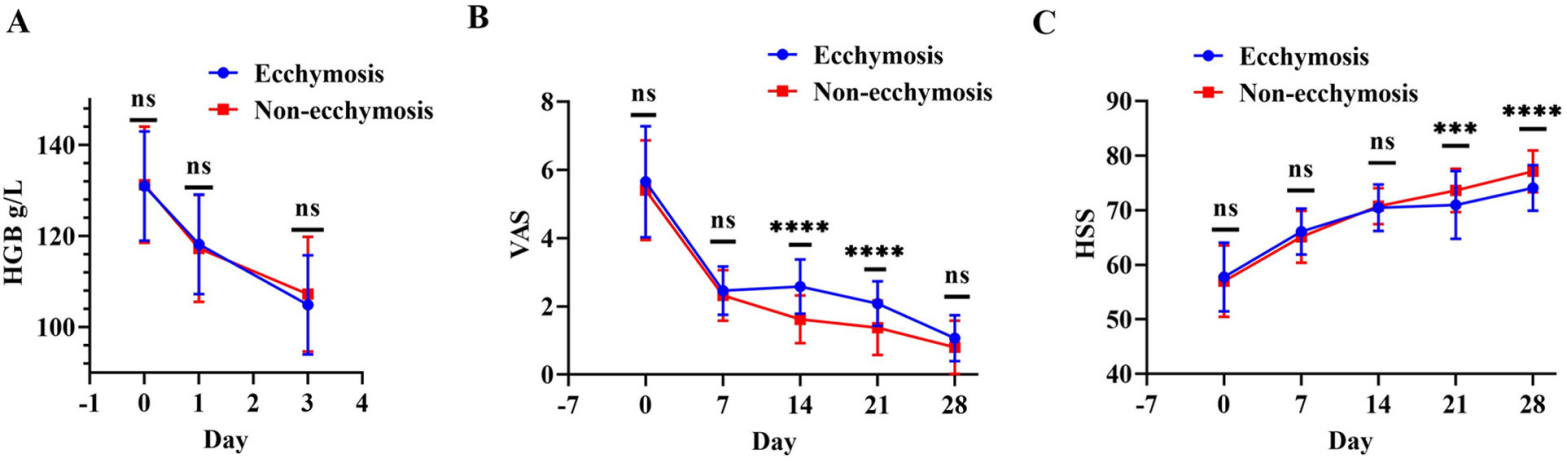
Comparison of postoperative changes in HGB, VAS, and HSS scores between the ecchymosis group and non-ecchymosis group. **(A)** Comparison of HGB levels; **(B)** comparison of VAS scores; **(C)** comparison of HSS scores.

There were no significant differences in preoperative and postoperative day 1 and 3 HGB changes between the ecchymosis and non-ecchymosis groups (*p* > 0.05) (Figure 3A). VAS scores did not significantly differ between the two groups preoperatively and on postoperative days 7 and 28 (*p* > 0.05); however, on postoperative days 14 and 21, the ecchymosis group had significantly higher VAS scores than the non-ecchymosis group (Figure 3B). Similarly, there were no significant differences in HSS scores between the two groups preoperatively and on postoperative days 1, 7, and 14 (*p* > 0.05); however, on postoperative days 21 and 28, the ecchymosis group showed significantly lower HSS scores compared to the non-ecchymosis group (Figure 3C).

In the SF-36 survey at 4 weeks postoperatively, the ecchymosis group exhibited significantly lower scores in both Physical Health Component (PHC) and Mental Health Component (MHC) dimensions compared to the non-ecchymosis group (*p* < 0.001). Further analysis revealed that the ecchymosis group had significantly lower scores in Physical Functioning (PF), Role limitations due to physical health (RP), Bodily pain (BP), General health (GH), Mental health (MH), Role limitations due to emotional problems (RE), Social functioning (SF), and Health change (*p* < 0.05). There was no statistically significant difference in energy levels between the two groups (*p* = 0.11) (Table 2).

**Table 2:** Subscale scores of SF-36 among the two cohorts at 4weeks after operation.

Subscale	Total (*n* = 138)	Ecchymosis (*n* = 60)	Non-ecchymosis (*n* = 78)	*P*-value^a^
Physical health composite (PHC)	50.25 ± 11.94	43.94 ± 11.96	55.11 ± 9.44	<0.001^a^
Mental health composite (MHC)	61.48 ± 18.15	53.77 ± 20.49	63.37 ± 13.52	<0.001^a^
Physical functioning (PF)	52.57 ± 14.49	47.58 ± 15.22	56.41 ± 12.71	<0.001^a^
Role limitations due to physical health (RP)	25.00 ± 26.16	18.75 ± 24.63	29.81 ± 26.44	0.01^b^
Bodily pain (BP)	65.72 ± 13.94	56.57 ± 12.53	72.76 ± 10.49	<0.001^a^
General health (GH)	71.21 ± 13.67	52.85 ± 6.90	61.45 ± 5.19	<0.001^a^
Mental health (MH)	44.44 ± 38.91	66.13 ± 14.27	75.12 ± 11.87	<0.001^a^
Role limitations due to emotional problems (RE)	64.09 ± 18.91	31.67 ± 44.44	54.27 ± 30.91	<0.00^b^
Social Functioning (SF)	66.09 ± 18.91	52.96 ± 17.76	72.65 ± 14.95	<0.001^a^
Vitality (VIT)	66.09 ± 11.31	64.33 ± 12.70	67.44 ± 9.99	0.11^a^
Health change	90.76 ± 12.11	86.67 ± 12.58	93.91 ± 10.80	<0.001^a^

a Independent-samples *t*-test.

b Mann–Whitney *U* test.

Subgroup analyses stratified by age (≥70 vs. <70 years) revealed that older patients had a higher incidence of ecchymosis (55.2% vs. 34.1%, *p* = 0.02). In a binary logistic regression model incorporating age, gender, side of knee, medical history, surgical details, and laboratory findings, analysis of risk factors for post-TKA ecchymosis indicated that age was a significant risk factor for the occurrence of ecchymosis post-TKA, with an odds ratio (OR) of 1.25 (95% confidence interval: 1.02–1.53), *p* = 0.036 (Table 3).

**Table 3:** Potential risk factors of ecchymosis after TKA.

Risk factor	B	OR	95% CI	*p*-value
Age	0.22	1.25	1.02–1.53	0.036
Gender	−1.83	0.16	0.15–1.72	0.131
BMI	0.15	1.16	0.97–1.39	0.103
Surgical side	−3.62	0.03	0.00–1.17	0.060
Ever-smoker	0.45	1.56	0.26–9.34	0.626
Alcohol consumption	−2.14	0.12	0.01–1.74	0.119
Diabetes	0.64	1.90	0.59–6.18	0.284
Hypertension	−1.19	0.30	0.06–1.47	0.139
K-L score	4.71	111.65	0.76–16,519.53	0.064
Osteoporosis	2.74	15.41	0.95–249.16	0.054
Operative time	0.01	1.01	0.98–1.05	0.573
Tourniquet time	0.07	1.08	0.99–1.17	0.071
Intraoperative blood loss	−0.00	1.00	0.99–1.01	0.559
Preoperative HGB	−0.06	0.95	0.88–1.01	0.110
Preoperative PT	−27.854	0.00	0.00–6.27	0.066
Preoperative FIB	0.09	1.09	0.92–1.20	0.299

BMI, body mass index; K–L, Kellgren–Lawrence; HGB, hemoglobin; PT, prothrombin time; FIB, fibrinogen.

## Discussion

The effectiveness of TKA is undeniable, as it demonstrates satisfactory results in traditional surgical indicators such as ligament stability, joint mobility, and longevity of prosthetic use. However, up to 20% of patients express dissatisfaction with the surgical outcome (3). This discrepancy arises from the fact that traditional surgical indicators primarily evaluate the efficacy of the surgery from the perspective of the surgeon, overlooking and underestimating the true feelings and expectations of patients. Patients expect TKA to restore their ability to work and lead a normal life (15). From the patient's perspective, it is becoming increasingly important to supplement traditional indicators with patient-reported treatment outcomes to assess the postoperative health-related quality of life (HRQOL), which has evolved into a crucial goal of orthopedic surgery and a key measure of surgical success (16). Limited research has focused on the impact of post-TKA ecchymosis on patients' early QOL, function, pain, etc. This study, utilizing PROMs, for the first time, explores the influence of post-TKA ecchymosis on various aspects of patients' early QOL, function, and pain, while investigating the incidence and potential risk factors of post-TKA ecchymosis under routine anticoagulant therapy.

In this study, the average PHC and MHC scores of patients after TKA were 50.25 and 61.48, respectively. A multicenter study by Liu evaluating the SF-36 one year post TJA demonstrated PHC scores ranging from 59 to 74 and MHC scores ranging from 70 to 79 (17), slightly higher than our results. However, this study was conducted within one-month post-TKA, and as patients readjust to life, these indicators are expected to improve gradually, indicating overall satisfaction among patients undergoing TKA at our center.

The incidence rate of post-TKA ecchymosis in our study was 43.48%, which is higher than previously reported rates of around 33% (6). This discrepancy may be attributed to several factors. Firstly, the delayed onset of ecchymosis, with a median time of appearance at 3 days postoperatively, suggests that some cases may have been missed in previous studies where patients were discharged earlier. With the increasing adoption of enhanced recovery after surgery (ERAS) protocols, the trend towards same-day surgery has become more prevalent, potentially leading to under-reporting of ecchymosis in earlier studies (18). Secondly, the rigorous follow-up protocol in our study, which included weekly telephone surveys, may have captured more cases of ecchymosis that would have otherwise gone unnoticed in studies with less frequent follow-up. While direct comparisons with other studies are challenging due to differences in surgical protocols, anticoagulation regimens, and follow-up methods, our findings highlight the importance of considering the timing of ecchymosis onset and the impact of ERAS protocols on postoperative complications. Future studies with similar follow-up protocols and surgical environments are needed to further validate these findings.

It is widely acknowledged that VTE prevention post-TKA is essential, with guidelines for postoperative VTE prophylaxis in TJA widely employed by orthopedic surgeons, such as those developed by the American Academy of Orthopaedic Surgeons (AAOS) and the American College of Chest Physicians (ACCP) (19, 20). However, concern exists that routine anticoagulation may be excessive for some patients with relatively low VTE risk, as the AAOS and ACCP guidelines seemingly prioritize efficacy over safety (5). Moreover, given the significant reduction in surgical time attributable to surgical principles and equipment advancements in TKA, coupled with the rapid development of ERAS, post-TKA patients often commence early rehabilitation, including assisted weight-bearing exercises, thereby significantly lowering the risk of post-TKA VTE development. In this study, all patients received a routine 2-week anticoagulant regimen postoperatively, without personalized anticoagulation plans based on individual VTE risk levels, possibly contributing to the higher incidence of ecchymosis. Therefore, achieving a more optimal balance between post-TKA bleeding prevention and VTE prophylaxis remains paramount. Given the association between age and ecchymosis, older patients (≥70 years) may benefit from reduced anticoagulation intensity or shorter duration, balancing VTE prevention and bleeding risk.

This study demonstrates that the HRQOL of patients with ecchymosis following TKA is significantly reduced compared to those without ecchymosis, with differences all exceeding the MCID. The ecchymosis group achieved poorer results across both PHC and MHC dimensions, as well as within all seven subscales of the SF-36, indicating that post-TKA ecchymosis inflicts physical and psychological harm on patients. These findings are as anticipated, given that post-TKA patients are sensitive to limb issues, with ecchymosis generating a strong visual impact. This psychological implication may heighten patients' sensitivity to pain, as evidenced by significantly higher VAS pain scores in the ecchymosis group on postoperative days 14 and 21. The ensuing psychological distress and exacerbated pain contribute to a decline in early functional levels among post-TKA patients, as reflected in significantly lower HSS scores on postoperative days 21 and 28 in the ecchymosis group compared to the non-ecchymosis group. Similar to our results, Bierke et al. found that psychological factors influence the clinical outcomes following TKA for up to five years (21). Fortunately, most ecchymosis cases appear to be self-resolving, with almost ecchymosis in the ecchymosis group resolved by day 28 postoperatively. The median time to ecchymosis resolution (21–28 days) coincides with the critical early rehabilitation phase. Persistent ecchymosis may delay functional recovery or raise patient anxiety, necessitating tailored discharge counseling and follow-up schedules. Integrating information about ecchymosis into preoperative discussions and patient education to provide patients with an overview of the expected course of ecchymosis may help alleviate patient anxiety related to sudden-onset ecchymosis post-TKA. Additionally, with the advancement of medical technology, remote monitoring and robotic surgical systems offer new possibilities for improving postoperative care. Remote monitoring through applications has been demonstrated to effectively track patients' postoperative recovery (22). This technology enables real-time monitoring of patients' pain levels, mobility, and ecchymosis, thereby allowing for timely adjustments to treatment plans. Preoperative education should include visual aids (photographs of typical ecchymosis progression) and a timeline for resolution. For patients with ecchymosis, brief cognitive-behavioral interventions (2–3 sessions focused on anxiety management) could be integrated into postoperative follow-up.

Several studies have analyzed the risk factors for post-TKA ecchymosis. Kang et al. identified blood transfusions and occlusion of drainage tubes as risk factors for ecchymosis (9). Xiao et al. further studied patients routinely administered rivaroxaban as anticoagulation therapy, with their results indicating that age, BMI, history of hypertension, mean arterial pressure, albumin, and hemoglobin levels were independent risk factors for ecchymosis development (23). This study reveals age as a risk factor for post-TKA ecchymosis. With increasing age, there is a higher prevalence of joint surrounding tissue laxity, osteoporosis, increased vascular fragility, all potentially leading to increased occult blood loss post-TKA and subsequently elevating the incidence of ecchymosis. Hence, in the selection of anticoagulation plans for TKA patients, age as a risk factor should be taken into account to opt for more optimized protocols. Our subgroup analyses suggest that age and comorbidities may modulate ecchymosis risk, highlighting the need for personalized anticoagulation strategies in high-risk populations. In addition, variables such as osteoporosis showed borderline significance (*p* = 0.054), suggesting a potential association with ecchymosis that may require validation in larger cohorts. Future studies should prioritize these variables to clarify their clinical relevance.

This study possesses certain limitations. (1) It is a single-center study, thus inevitably subject to selection bias and researcher bias. (2) As an observational study, our findings are inherently limited in establishing causality between ecchymosis and postoperative outcomes. While we identified associations between ecchymosis and reduced QOL, pain, and function, the lack of intervention precludes definitive conclusions about optimal management strategies (e.g., anticoagulation adjustment or psychological support). Future randomized controlled trials are warranted to explore whether targeted interventions for ecchymosis can improve patient-centered outcomes. (3) The relatively small sample size (*n* = 138) may have limited the statistical power to detect subtle associations, particularly in multivariable analyses. Larger cohorts are needed to confirm our findings and validate risk factors with borderline significance. (4) While we controlled for demographic and surgical variables, unmeasured confounders (e.g., rehabilitation compliance) may influence outcomes. Future studies should incorporate objective measures of rehabilitation adherence (e.g., wearable activity trackers) to address this gap.

## Conclusions

Under routine anticoagulant management, the occurrence of ecchymosis post-TKA is relatively high. TKA patients with ecchymosis experience a significant early decline in QOL postoperatively, exacerbated pain, and an impact on knee function. Age emerges as a possible risk factor for early post-TKA ecchymosis. Incorporating relevant ecchymosis information into preoperative education and providing appropriate psychological interventions for patients with ecchymosis may be crucial. Optimization of post-TKA anticoagulant regimens necessitates further refinement.

## Data Availability

The raw data supporting the conclusions of this article will be made available by the authors, without undue reservation.
