# Correction: Quality of life reported by patients with ecchymosis following total knee arthroplasty

**DOI:** 10.3389/fsurg.2026.1883575

**Published:** 2026-06-10

**Authors:** Zhibing Gong, Hanglin Qiu, Huantang Zhang, Yanyan Xu, Rongkai Wu, Qianjin Zhang, Hanghui Lin, Zhaoke Wu, Fudong Xu, Zhikun Zhuang, Changyu Huang

**Affiliations:** Department of Orthopaedic Surgery, Quanzhou Orthopedic-Traumatological Hospital, Quanzhou, China

**Keywords:** total knee arthroplasty, quality of life, ecchymosis, patient-reported outcome measures, risk factors

Two numerical typographical errors were present in the first paragraph of the Results section, affecting the consistency of patient baseline descriptions. All data in Table 1 are correct and consistent with the original study dataset. The errors are limited to descriptive text and do not influence the study results, statistical analyses, or main conclusions.

A correction has been made to the section Results, first paragraph. This sentence previously stated:

“Of these participants, there were 8 cases of traumatic arthritis, 8 cases of rheumatoid arthritis, and 130 cases of osteoarthritis. There were 15 male and 123 female patients, with a mean age of 67.91 ± 7.24 years (range: 51–84), 83 cases involving the left knee and 75 cases involving the right knee, and a mean BMI of 25.57 ± 3.85 kg/m^2^.”

The correct sentence appears below:

“Of these participants, there were 8 cases of traumatic arthritis, 8 cases of rheumatoid arthritis, and 122 cases of osteoarthritis. There were 15 male and 123 female patients, with a mean age of 67.91 ± 7.24 years (range: 51–84), 63 cases involving the left knee and 75 cases involving the right knee, and a mean BMI of 25.57 ± 3.85 kg/m^2^.”

These corrections ensure consistency with the total sample size (*n* = 138) and Table 1.

The original version of this article has been updated.

